# Effect of Topical Capsaicin on Painful Sensory Peripheral Neuropathy in Patients with Type 2 Diabetes: A Double-Blind Placebo-Controlled Randomised Clinical Trial

**DOI:** 10.7759/cureus.11147

**Published:** 2020-10-25

**Authors:** Batakeh B. Agoons, Mesmin Dehayem Yefou, Jean-Claude Katte, Martine Claude Etoa Etoga, Dayawa D Agoons, Faustin Yepnjio, Anne Boli, Yves Wasnyo, Eugene Sobngwi, Jean-Claude Mbanya

**Affiliations:** 1 Internal Medicine, Bafang District Hospital, Bafang, CMR; 2 Diabetes and Endocrinology, Central Hospital, Yaounde, CMR; 3 Internal Medicine, Faculty of Health Sciences, University of Bamenda, Bamenda, CMR; 4 Public Health Sciences, Faculty of Medicine and Biomedical Sciences, University of Yaounde 1, Yaounde, CMR; 5 Internal Medicine, Faculty of Medicine and Pharmaceutical Sciences, University of Douala, Yaounde, CMR; 6 Medicine, University of Pittsburgh Medical Center Pinnacle Hospital, Harrisburg, USA; 7 Neurology, Central Hospital, Yaounde, CMR; 8 Internal Medicine, Faculty of Medicine and Biomedical Sciences, University of Yaounde 1, Yaounde, CMR; 9 Diabetes and Endocrinology, Central Hospital, Yaounde, Yaounde, CMR; 10 Emergency, Central Hospital, Yaounde, Yaounde, CMR

**Keywords:** capsaicin, peripheral neuropathy, type 2 diabetes, randomised clinical trial

## Abstract

Objective

The aim of this study was to evaluate the efficacy of capsaicin in inducing significant pain relief in a population of sub-Saharan African type 2 diabetic patients with painful peripheral neuropathy.

Design

This was a prospective double-blind placebo-controlled randomised clinical trial.

Setting

A single tertiary-level hospital diabetes center in Yaounde, Cameroon.

Participants

Twenty-two participants with type 2 diabetes mellitus, presenting with painful diabetic neuropathy, aged 18 years and above.

Intervention

Participants were equally randomised to capsaicin or placebo. Each drug was to be applied on the lower limbs thrice daily. Follow-up was done every two weeks for eight weeks.

Main outcome measure

Reduction in the median pain score from baseline, as assessed by the Visual Analogue Scale.

Results

Twenty-two participants aged 57.5 (50-60) years with a median pain intensity of 6.8 units in the capsaicin group and 5.8 units in the placebo group were included; at inclusion, there was no significant difference in the two groups (p=0.29). After two weeks, the value of pain intensity was 3.3 [2.5-4.0] vs 5.0 [4.0-7.4] (p=0.003); at week four, 3.0 [2.5-3.3] vs 5.0 [4.2-5.5] (p=0,02); at week six, 3.3 [2.5-4.0] vs 4.8 [4.0-6.0] (p=0.03); and at week eight, 6.6 [6.0-7.0] vs 5.2 [5.0-6.0] (p=0.54) for capsaicin and placebo respectively.

Conclusion

This study, carried out due to a paucity of information on the effect of capsaicin and painful diabetic neuropathy in sub-Saharan African diabetes patients, shows that capsaicin significantly reduces neuropathic pain with worsening after eight weeks of use.

Trial registration number

Pan Africa Trial Registry: PACTR202003714748946.

## Introduction

The treatment of painful diabetic neuropathy, according to the Ad Hoc Panel on Endpoints for Diabetic Neuropathy Trials management is firstly, to assure optimal glucose control, secondly, to symptomatically treat the pain, and thirdly, with the use of ancillary therapies directly interfering with the pathophysiologic cycle of diabetic neuropathy [1]. Symptomatic therapies for painful diabetic neuropathy include tricyclic antidepressants, anticonvulsants, gamma-aminobutyric acid analogues, selective serotonin reuptake inhibitors, and rarely, opioids [2]. Despite this seemingly rich availability of options, the effective treatment of painful diabetic neuropathy remains a challenge for both physicians and patients [3]. Most of these drugs are orally administered, and thus present with a high risk of systemic side effects and decreased drug bioavailability due to hepatic first-pass mechanism [4].

Capsaicin (trans-8-methyl-N-vanillyl-6-nonenamide) is the active ingredient found in various species of chili peppers and exhibits pharmacologic effects on type C nociception nerve fibers, which are necessary for the conduction of slow neuropathic pain [5]. Repeated application of topical capsaicin drug formulation causes functional injury to peripheral nerves, resulting in desensitization to painful stimuli [6]. This peculiar characteristic of capsaicin is the basis for its usage in relieving the pain of HIV neuropathy, post-herpetic neuralgia and surgical postoperative pain [7]. Topical capsaicin has also demonstrated moderate efficacy in diabetic peripheral neuropathy in studies conducted in Caucasians [8-11]. Despite the anatomy and physiology of a nerve being the same in all populations, pain perception and response to treatment show ethnic and racial differences with treatment response in Africans different from Caucasians [12]. Due to little available data on the long-term efficacy of topical capsaicin in the reduction of painful sensory diabetic neuropathy in sub-Saharan patients with type 2 diabetes, we sought to determine if pain relief induced by capsaicin is significant enough to be experienced by this population type. We, therefore, aimed to investigate the long-term efficacy of topical capsaicin in the reduction of neuropathic pain in type 2 diabetes patients diagnosed with diabetic neuropathy in a clinical setting in Cameroon. 

## Materials and methods

Ethics

The study protocol was approved by the Institutional Review Board (IRB) of the Faculty of Medicine and Biomedical Sciences (0055/UY1/FMSB/VDRC/CSD), University of Yaoundé 1, Cameroon and the Centre Regional Ethics Committee for Research in Human Health (0076/CRERSHC/2018). All study procedure was done in accordance with the 2013 revised Helsinki Declaration and the Good Clinical Practice guidelines of the International Conference on Harmonisation. All participants provided a written informed consent form before enrollment into the study. This study was also retrospectively registered on the Pan African Clinical Trial Registry with the unique identification number PACTR202003714748946.

Study design and setting 

This was a double-blinded placebo-controlled randomised clinical trial conducted from the 15th of January 2018 to the 31st of May 2018 (the last participant enrolled on the 2nd of April 2018) at the National Obesity Center, Yaoundé Central Hospital, Cameroon. 

Study participants

Participants were assessed for eligibility on the basis of presenting complaints consistent with painful diabetic neuropathy at diabetes out-patient consultation. All participants who had daily pain or painful paresthesias in a neuropathic or radiculopathic distribution with an intensity between 4 and 7 on a Visual Analogue Scale instrument (VAS, Schlenker®, Lombard, USA) interfering with daily activities, work, or sleep for at least three months were invited to participate in the study. The Visual Analogue Scale is a valid and practical instrument to determine pain intensity in clinical settings [13-15]. Pain is divided into three categories based on VAS scores; Mild (0.5-4.4), Moderate (4.5-7.4), and Severe (7.5-10) [16]. Concerns with respect to recruiting subjects with severe pain intensity and an implied poor quality of life state, and potentially putting a section of them on placebo for eight weeks were raised for ethical reasons. As such, we limited recruitment to those with moderate pain intensity. 

We excluded participants with other known probable cause of peripheral neuropathy such as HIV/AIDS, megaloblastic changes on blood smear, history of allergies to any capsaicin product, alcohol consumption history, presence of open skin lesions at the site of application of study medication, signs of local infection on limbs or amputations, pregnant or lactating females, other topical medication at the site of application of study drug and all eligible patients who did not give a signed written informed consent. 

Randomisation and blinding

Randomisation was done using computer software (Random Allocation Software version 2.0, Mahmood Saghaei, Isfahan, Iran), with a 1:1 varying blocking type to preserve internal validity. The block size used was 2 and 4. Thus, randomisation divided the eligible candidates into two groups, capsaicin group and placebo group, equally. Generation of random allocation sequence, enrolment of participants and distribution of the drug was done by different trained workers. The study drug (capsaicin 0.075% gel) and the placebo drug (miconazole cream) were in identical no-label white tubes. Both drugs were odourless and were dispensed to participants according to randomised group assignment by a study pharmacist who was not involved in the generation of the randomisation sequences. 

Procedures and intervention 

Painful diabetic neuropathy was our condition of interest in this study. As such, two questionnaire/score types were used to diagnose this. First, we sought to determine if a patient had peripheral neuropathy. This was done using the Toronto Neuropathy Score. For all diagnosed participants with neuropathy, the Douleur Neuropathique Score was used to determine pain, being the primary/major symptom. Thus, painful distal sensory neuropathy was diagnosed using the Toronto Neuropathy Score [17] and the Douleur Neuropathique 4 (DN4) questionnaire [18,19]. Hepatitis and HIV serology tests were done to exclude these causes. A full blood count and smear were done in all participants, and the absence of megaloblastic changes/macrocytosis eliminated cobalamin and folate deficiencies as possible causes of peripheral neuropathy.

At baseline, a complete physical examination was performed including the recording of biophysical parameters (blood pressure, weight, and height). A short interview using a pre-tested questionnaire was used to capture socio-demographic characteristics, history of diabetes, characteristics of the pain, the effect of pain on quality of life and on-going anti-diabetic treatment

To reduce bias, randomisation, drug (capsaicin and placebo) sharing and follow-up were done by different groups of trained clinical workers. Blinding was assured by separating the staff members who performed eligibility screening/physical exam from those who carried out subject follow up and those who measured the outcomes. The clinical research team responsible for data collection carried out questioning on the basis of a pre-set questionnaire.

Participants were required to apply the prescribed drugs topically on the feet three times daily. Evaluation of the participants including assessment of the pain severity, assessment of the quality of life, vital signs and examination, and questioning regarding the adverse effects were performed at every two-week follow-up visit. No questions which could reveal drug concealment such as drug texture and form were asked. Compliance was assessed by direct questioning. 

Data monitoring and safety

A data and safety monitoring board was set up and could decide to withdraw a study participant if there was development of any serious harm as a result of the trial. The board was accessible by telephone to all participants throughout the study. At the end of the eighth week, the study was stopped. Equipoise was assured by making sure all investigators, participants and data analysts were blinded to the allocation. Only members of the data and safety monitoring board were aware of the participants per allocated arm.

Study outcome measures

The primary outcome/endpoint of the study was the reduction in the median pain score from baseline, as assessed by the Visual Analogic Scale (0-10 points). The secondary endpoint was the assessment of the level of improvement in the quality of life score after the intervention. This was evaluated using the Physician’s Global Evaluation Score. This scale has been used to determine the improvement of quality of life in patients suffering from eczematous dermatoses [20] and diabetic neuropathy [21].

Sample size 

The sample size was estimated on the basis of the proportion of patients to experience pain relief on a valid pain intensity measuring scale as the main outcome of interest. Using the formula for clinical trial sample size calculations using proportions [22], and percentage of pain relief for capsaicin and placebo at 0.73 and 0.21 respectively, according to a previous study [21], with α set at 5%, β at 20%, and power of 80%, we obtained a minimum value of eight participants per group. We later included 11 patients per group.

Statistical analysis

The primary analysis was intention-to-treat and involved all patients who were randomly assigned. We used non-parametric test (Man Whitney U test) to compare the median pain score from the Visual Analogue Scale between the two treatment groups. Values were expressed as median (interquartile range) and frequency (percentages) were necessary. Data were analysed using the Statistical Package for Social Sciences (SPSS) version 23.0 (IBM Corp, Armonk, USA). Statistical significance was set at p≤ 0.05.

## Results

Study participants

**Figure 1:**
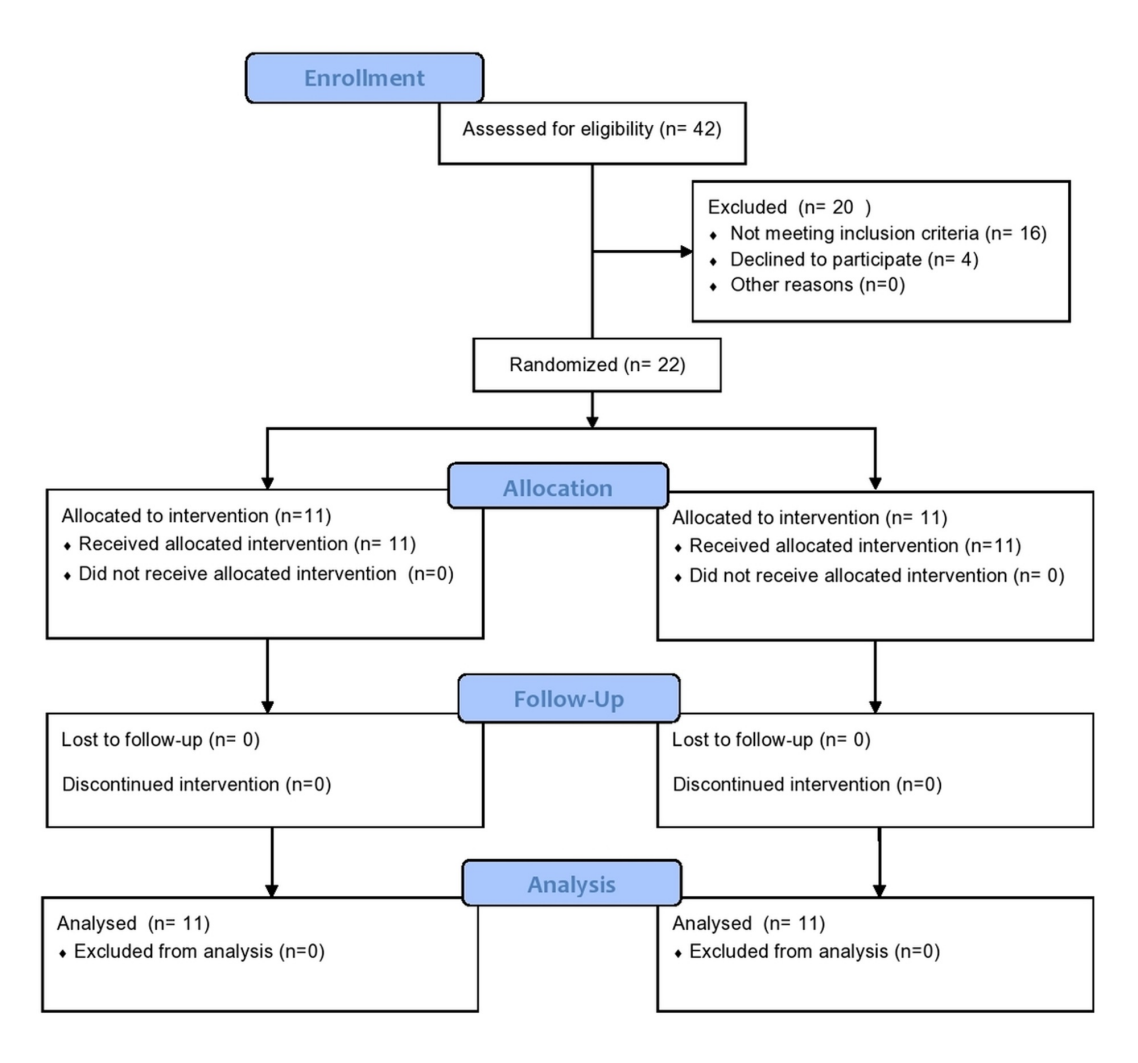
Participant flow chart

Figure 1 shows the study flow diagram. We screened 42 eligible participants and 20 were excluded. The 22 participants who met the eligibility criteria were randomised; 11 participants were assigned to receive topical capsaicin and 11 participants to placebo. All study participants completed the eight weeks of treatment. 

Baseline characteristics of the patients

The median age of participants in the study was 57.5 (50-60) years, and 12 out of 24 of the patients were men. There were no significant between-group differences in the demographic and baseline clinical and biophysical characteristics. These baseline characteristics are shown in Table 1. 

**Table 1:** Demographic and clinical characteristics of patients at baseline

Characteristics	Total (N=22)	Capsaicin Group (N=11)	Placebo Group (N=11)
Age, median (IQR)	57.5 (50-60)	56.0 (50-60)	58.0 (50-62)
Sex, n (%)			
Male	12	7 (64)	5 (45)
Female	10	4 (36)	6 (55)
Diabetes duration, years	7.5 (5-12)	9.0 (6-12)	5.0 (4-10)
Diabetes treatment			
Oral anti-diabetic	11	05	06
Insulin	6	03	03
Both	6	03	03
Self-reported hypertension	17	08	09
BMI kg/m^2^	31.6 (29.4-37.2)	32.0 (23.1-37.3)	31.2 (29.3-39.5)
Systolic BP mmHg	133.5 (120-145)	132 (102-145)	135 (122-148)
Diastolic BP mmHg	80 (73-90)	85.0 (70-90)	78.0 (77-88)
Triglycerides g/l	1.1 (0.8-1.75)	1.0 (0.6-1.5)	1.5 (0.8-2.1)
Total cholesterol g/l	1.5(1.3-2.0)	2.5 (1.7-2.5)	1.8 (0.76-2.6)
HbA_1_C %	6.2 (5.5-7.2)	6.8 (5.5-7.4)	6.2 (5.4-6.2)

Primary outcome: reduction of median pain intensity values

**Figure 2:**
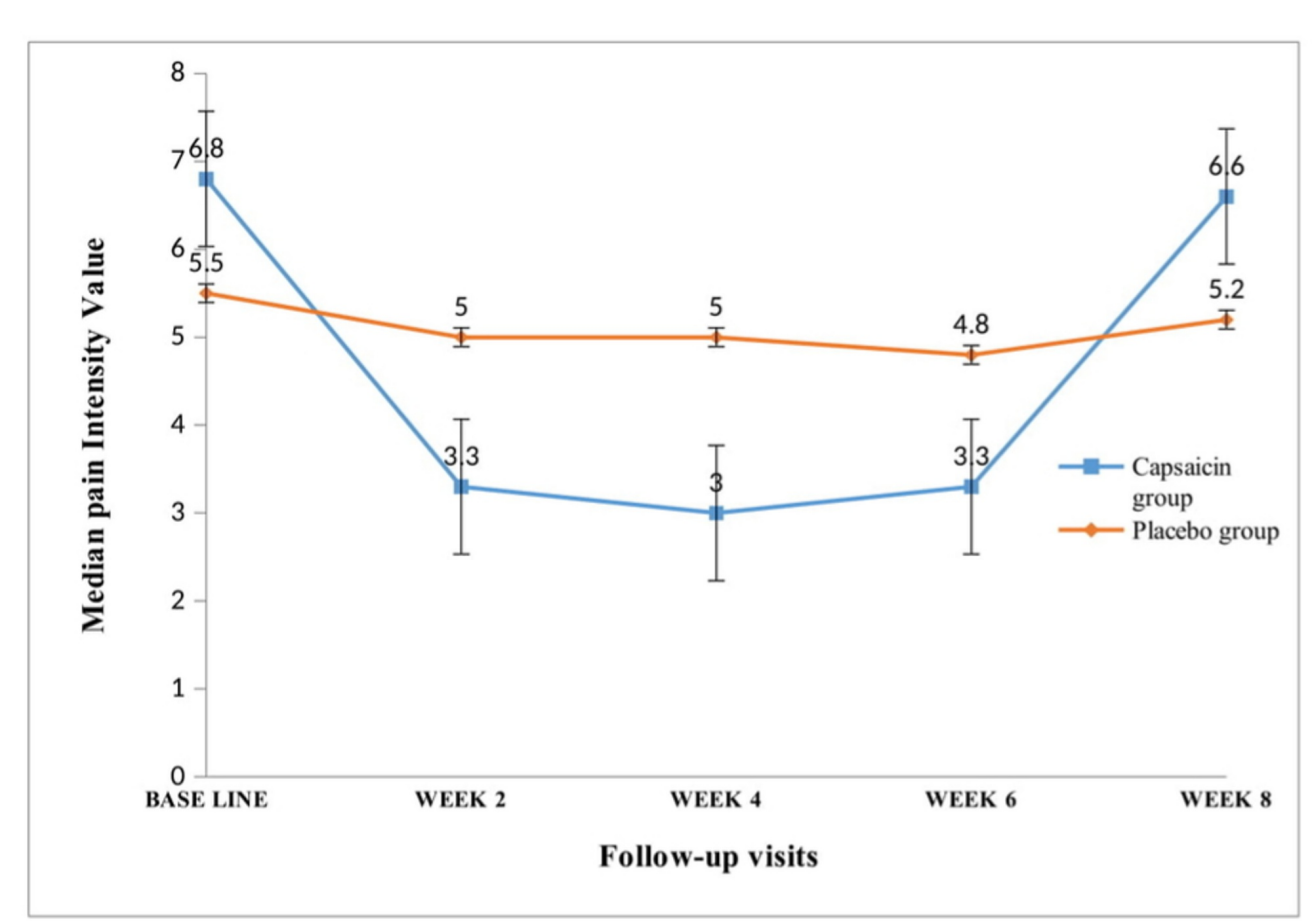
Evolution of median pain values per two weeks in both treatment arms

Figure 2 shows the results of the effect of our intervention on the pain intensity values for each group from baseline to the endpoint of the study (eight weeks). There was a significant reduction in the median pain scores in the capsaicin group compared to the placebo group after two weeks of treatment (3.3 (2.5-4.0) vs 5.0 (4.0-7.4), p=0.003), which was maintained to the sixth week of treatment (3.3 (2.5-4.0) vs 4.8 (4.0-6.0), p=0.03). At the eighth week of treatment, there was a rebound in the median pain scores in the capsaicin group and there was no longer a difference between the capsaicin group and the placebo group (6.6 (6.0-7.0) vs 5.2 (5.0-6.0), p=0.54). 

Secondary outcome: improvement of quality of life score

Quality of life analysis was done at the fourth and eighth weeks of the study. At the fourth week of the intervention, nine participants taking capsaicin attested to a positive change, with five feeling “much better”, and four feeling “moderately better”. At the eighth week, however, seven participants in the capsaicin group felt “much worse” compared to baseline. Comparison for both groups revealed no statistically significant improvements as shown in Table 2.

**Table 2:** Quality of life score/analysis

Quality of Life	At Fourth Week		At Eighth Week	
	Capsaicin group (N=11)	Placebo group (N=11)	Capsaicin group (N=11)	Placebo group (N=11)
Much better	4	0	0	0
Moderately better	5	5	0	2
No Change	2	4	4	6
Worse	0	2	3	3
Much worse	0	0	4	0

Safety 

Of the 11 participants allocated to receive the drug, eight complained of a burning, stinging sensation at the site of application only. Two participants, in addition to this burning sensation, complained of sneezing and tearing eyes upon administration, and another participant complained of diffuse redness on site of application, in addition to the burning. These side effects were noticed upon first administration and reduced significantly or were absent by the second week of treatment. Treatment was not interrupted. The eleven participants assigned to the placebo group had no side effects throughout the duration of study as shown in Table 3.

**Table 3:** Table showing adverse effects due to treatment in both groups

Adverse Effects Noted	Capsaicin n (%) N=11	Placebo n (%) N=11
Burning only		
At week 2	11 (100)	0
At week 4	0 (0)	0
At week 6	0	0
At week 8	8 (72.7)	0
Burning/sneezing/tearing eyes		
At week 2	2 (18.2)	0
At week 4	0	0
At week 6	0	0
At week 8	0	0
Burning and redness		
At week 2	1 (9.1)	0
At week 4	0	0
At week 6	0	0
At week 8	0	0

## Discussion

We do not know of any other similar study involving capsaicin for painful diabetic polyneuropathy in Africa. As such, the results presented by this study are of particular interest for health professionals in this area. The results show that topical capsaicin is efficacious in pain reduction as from two weeks and up to six weeks after the commencement of treatment. This therapeutic benefit was lost after six weeks given that there was no difference in the median pain scores between the capsaicin group and the placebo group at the study endpoint (eight weeks of treatment). These findings suggest that topical capsaicin may be used alone as treatment for painful distal sensory diabetic neuropathy for up to six weeks and that other agents may be added to provide pain suppression in longer term treatment strategies. 

Capsaicin is known to mediate its effect by causing the de-functionalization of the C fiber nociceptors (neurolysis). It is a TRPV1 (transient receptor potential vanilloid 1) agonist and its prolonged activation of TRPV1 results in loss of receptor functionality, causing impaired local nociception for extended periods [23]. Also de-functionalization of peripheral nerve fibers is partially as a result of capsaicin-induced substance P depletion, along with other sensory mediators (calcitonin gene-related peptide) in the spinal dorsal root ganglia. Regeneration or re-innervation of these nerve fibers after a functional insult is completed after about six to eight weeks [23], which may explain the increase in the pain intensity after six weeks of treatment in this study. 

There was an improvement in the quality of life at week four of the participants in capsaicin group, manifested by better sleep and less interference in physical activities and emotion. Nine out of 11 participants in the capsaicin group experienced an improvement in their quality of life. This improvement of the quality of life seen after four weeks of treatment could be due to the improvement in pain scores seen during the first six weeks of treatment. The quality of life of participants in the capsaicin group worsened at the eighth week most likely due to the worsening pain intensity due to nerve re-innervation.

Our study also sought to identify the safety profile of topical capsaicin. All the 11 participants undergoing treatment complained of a burning or stinging sensation at site of application of drug after the onset of treatment. However, these sensations reduced or disappeared after continuous use of the drug by the second week of treatment. This finding corresponds to capsaicin-induced initial hyperalgesia of nociceptors followed by de-sensitization [24]. One participant out of the 11 complained of an on-site burning pain associated with erythema. This redness may be attributed to the release of histamine from mast cells also seen with topical capsaicin treatment [6].

Our trial has some limitations to be taken into consideration. The absence of standard nerve conduction studies (best methods in diagnosing and determining the type of neuropathy) may have improved the clinical relevance to the current study. However, in many resource-limited settings, these neuro-electrical tests are not available and so clinicians rely on short and validated tools such as the DN4 Questionnaire, Toronto Clinical Neuropathy score and the Visual Analogue Scale in the assessment of neuropathic pain and pain intensity respectively. The small sample size did not permit us to examine for significance of the quality of life scores in this study. 

## Conclusions

Topical capsaicin clinically improves neuropathic pain in Sub-Saharan type 2 diabetes patients with painful distal sensory neuropathy after two weeks and up to six weeks after the commencement of treatment. Hence, other pain-relieving drugs may be considered as an adjuvant therapy to topical capsaicin for long-term reduction of pain in patients with type 2 diabetes with painful distal sensory polyneuropathy. 
